# The Vernix Caseosa is the Main Site of Dioxin Excretion in the Human Foetus

**DOI:** 10.1038/s41598-017-00863-9

**Published:** 2017-04-07

**Authors:** Seiichi Morokuma, Kiyomi Tsukimori, Tsuguhide Hori, Kiyoko Kato, Masutaka Furue

**Affiliations:** 1 Department of Obstetrics and Gynecology, Kyushu University Hospital, Fukuoka, Japan; 2 Department of Obstetrics, Fukuoka Children’s Hospital, Fukuoka, Japan; 3 Fukuoka Institute of Health and Environmental Science, Fukuoka, Japan; 4 Department of Obstetrics and Gynecology, Graduate School of Medical Sciences, Kyushu University, Fukuoka, Japan; 5 Research and Clinical Center for Yusho and Dioxin, Kyushu University Hospital, Fukuoka, Japan; 6 Department of Dermatology, Graduate School of Medical Sciences, Kyushu University, Fukuoka, Japan

## Abstract

Dioxins are highly toxic to foetuses and prenatal exposure leads to adverse health effects; however, the metabolic pathways involved in dioxin excretion are poorly understood. We determined the dynamics of maternal-to-foetal dioxin transfer during normal pregnancy and how foetuses eliminate polychlorinated dibenzo-*p*-dioxins, polychlorinated dibenzofurans, and non-*ortho* polychlorinated biphenyls. Dioxin levels in maternal blood, cord blood, placenta, vernix caseosa, meconium, and amniotic fluid were analysed by high-resolution gas chromatography/mass spectrometry. The average levels of total dioxins, expressed as picograms of toxic equivalency quantity per gram of lipid and in parentheses, dioxin fraction, with maternal blood levels arbitrarily set as 100%, were as follows: maternal blood, 15.8 (100%); placenta, 12.9 (81.5%); cord blood, 5.9 (37.2%); vernix caseosa, 8.4 (53.2%); **m**econium, 2.9 (18.2%); and amniotic fluid, 1.5 (9.2%). Similar proportions were observed for each dioxin congener. Thus, the highest content of foetal dioxins was observed in the vernix caseosa, indicating that this is the major site of dioxin excretion in human foetuses.

## Introduction

Prenatal exposure to dioxins can result in various adverse health effects^1^. Yusho disease is caused by exposure to rice oil contaminated with non-*ortho* polychlorinated biphenyls (PCBs), polychlorinated dibenzofurans (PCDFs), and polychlorinated dibenzo-*p*-dioxins (PCDDs). Studies of patients with Yusho disease have revealed increased miscarriage frequency, premature birth, and foetal death; a significant negative correlation between the maternal blood dioxin level and birth weight; and an increased risk of developing hyperpigmentation in the new-born in conjunction with elevated maternal blood dioxin level^1–3^. Although the transfer of dioxins from mother to foetus has been reported in humans^4, 5^, little is known regarding their distribution in and elimination from the human foetus.

A previous study, in which we assessed dioxin levels in maternal blood, placenta, and cord blood^6^, demonstrated that 1) the dioxin level in cord blood is approximately half of that in maternal blood, 2) congeners with a high toxic equivalency factor (TEF) value, such as 1,2,3,7,8-penta CDD and 2,3,4,7,8-penta CDF, are easily transferred from maternal blood to the placenta, and 3) PCDDs display an enhanced capacity for maternal blood-to-foetus transfer through the placenta as compared to PCDFs and non-*ortho* PCBs. However, the metabolic pathways responsible for dioxin excretion in the human foetus are not fully understood.

To date, cord blood is mainly used to detect foetal exposure to dioxins. However, dioxins in the blood are in a dynamic state of metabolism, tissue distribution, and excretion, and their levels in the blood may not represent the true extent of exposure^7^. Meconium is a matrix that has been previously analysed to detect exposure to a number of xenobiotic agents^8, 9^. In adults, the faeces and sebum are important discharge sources of dioxins^10, 11^. It is thought that the material discharged from the foetus, including meconium and vernix caseosa, directly reflects the quantity of dioxins. However, the vernix caseosa has not been examined.

This pilot study aimed to determine the dynamics of maternal-to-foetal dioxin transfer, including vernix caseosa, during normal pregnancy.

## Results

**Figure 1:**
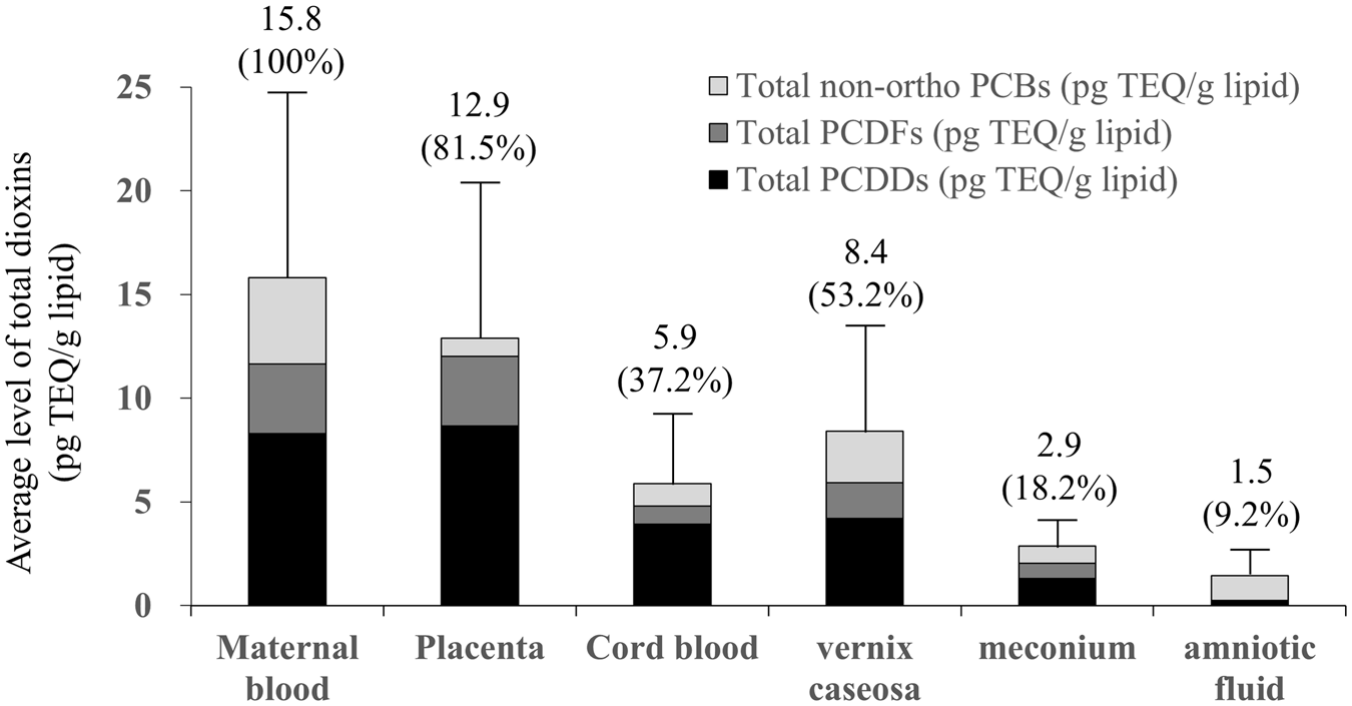
Average levels and SDs of total dioxins. Values in brackets indicate the proportion relative to maternal blood, which was set as 100%. TEQ, Toxic equivalent quantity; SD, standard deviation.

The average levels of total dioxins in each of the sampled tissues are shown in Fig. 1. The average levels ± SDs of PCDDs (pg TEQ/g lipid) were as follows: maternal blood, 8.3 ± 5.2 (100%); placenta, 8.7 ± 5.2 (104.5%); cord blood, 3.9 ± 2.6 (47.5%); vernix caseosa, 4.2 ± 2.8 (50.9%); meconium, 1.3 ± 1.1 (15.9%); and amniotic fluid, 0.2 ± 0.5 (2.9%). The average levels ± SDs of PCDFs (pg TEQ/g lipid) were as follows: maternal blood, 3.4 ± 2.2 (100%); placenta, 3.4 ± 2.0 (99.6%); cord blood, 0.9 ± 0.6 (26.0%); vernix caseosa, 1.7 ± 1.5 (50.7%); meconium, 0.72 ± 0.4 (21.4%); and amniotic fluid, 0.01 ± 0.04 (0.4%). The average levels ± SDs of non-*ortho* PCBs (pg TEQ/g lipid) were as follows: maternal blood, 4.1 ± 2.5 (100%); placenta, 0.9 ± 0.5 (20.9%); cord blood, 1.1 ± 0.8 (25.8%); vernix caseosa, 2.5 ± 1.4 (59.8%); meconium, 0.8 ± 0.5 (20.1%); and amniotic fluid, 1.2 ± 1.1 (29.1%). Similar proportional values were observed for each dioxin congener. The highest content of dioxins in foetuses was observed in the vernix caseosa, followed in descending order by cord blood, meconium, and amniotic fluid.

The detection rate of dioxin congeners was 51% in maternal blood, 50% in umbilical cord blood, 62% in the placenta, 42% in the meconium, 53% in the vernix caseosa, and 13% in the amniotic fluid. PCDDs of either congener were not detected in nine amniotic-fluid samples. Similarly, PCDFs of either congener were not detected in two meconium and twelve amniotic-fluid samples. Regarding non-*ortho* PCBs, none of the congeners was detected in three amniotic-fluid samples. However, all other congeners were detectable.

## Discussion

In the present study, we observed that the level of dioxins in cord blood was approximately 40% of that in maternal blood. However, the dioxin level in the foetal vernix caseosa was approximately 50% of the maternal blood level, indicating that the vernix caseosa constitutes an integral part of the dioxin excretion system in the human foetus. The weight and fat content of the vernix caseosa and meconium are approximately 5 g and 20–40% and 60–200 g and 2–3%, respectively. Therefore, the amount of fat of the vernix caseosa is approximately half that of the meconium. However, the level of dioxins per gram lipid in the vernix caseosa was more than twice that in meconium, indicating that dioxins are preferentially excreted into the vernix caseosa.

The vernix caseosa and meconium in the new-born are excellent depositories for persistent pesticide residues and hence can provide a historical record for prenatal exposure to these residues^9^. Therefore, these excrements are considered ideal matrices for analysing foetal exposure to various environmental toxins.

The vernix caseosa is a protective layer covering the foetus during the last trimester and is rich in cholesterol, free fatty acids, and ceramides^12^. Considering the highly lipophilic nature of dioxins^13–15^, it is logical to hypothesize that the inside-to-outside excretion of dioxins via lipid-rich epidermis, sebum, and the vernix caseosa may constitute a critical pathway enabling the foetus to lower the internal concentration of dioxins. Similar trans-epidermal excretion of dioxins has been previously described: high levels of dioxins were detected in the sebum of patients with Yusho disease and Yucheng disease (dioxin intoxication)^15^, and a study involving the Ukrainian President, who was exposed to highly concentrated tetrachlorodibenzo-*p*-dioxin, demonstrated the excretion of highly-concentrated dioxins in his sebum^14^. To the best of our knowledge, this study is the first to demonstrate that the vernix caseosa is one of the main sites of dioxin excretion in the human foetus.

We conclude that analysis of the vernix caseosa can detect antenatal foetal exposure to environmental toxins, specifically, heavy metals and pesticides. Vernix caseosa analysis is gaining acceptance in the scientific and medical communities as it comes with several advantages, such as provision of *in utero* record of exposure dioxins, non-invasiveness, and easy sample collection. However, as this pilot study was limited by its small sample number, no definite conclusions can be drawn, and future examination is necessary.

## Methods

### Study subjects and sampling

We followed the pregnancy and childbirth of 16 Japanese women from October 2009 to February 2011. The mean age at delivery was 31.8 ± 5.8 years (range, 20–42 years). Two subjects had a vaginal delivery and 14 had a caesarean section. The mean pre-pregnancy BMI and BMI at delivery were 21.5 ± 3.6 (range, 17.3–27.7) and 25.1 ± 3.1 (21.9–29.1), respectively. The mean gestational age at delivery was 38.1 ± 1.1 weeks (range, 36.9–41.0 weeks). The mean birth weight was 2995.8 ± 496.8 g (range, 2380–4135 g). Apgar scores at 1 and 5 min were 8.3 ± 0.6 (range, 7–9) and 8.9 ± 0.2 (8–9), respectively.

Amniotic fluid (20 mL) was obtained during delivery. Maternal blood (20 mL), cord blood (20 mL), placenta (20 g), vernix caseosa (5 g), and meconium (5 g) were obtained immediately following delivery. All specimens were stored in glass containers at −20 °C until analysis.

All experimental protocols were designed according to the Declaration of Helsinki’s ethical principles, were performed in accordance with the Ethical Guidelines for Medical and Health Research Involving Human Subjects, and were approved by the Institutional Review Board of Kyushu University. Prior informed consent was obtained from all participants.

### Analytical methods

After the extraction of lipids from the biological samples, the extracts were concentrated to dryness, and lipid contents were determined gravimetrically. PCDD, PCDF, and non-*ortho* PCB levels in the samples were analysed by high-resolution gas chromatography/mass spectrometry. The gas chromatograph used was an Agilent 7890 A (Agilent Technologies Inc., Palo Alto, CA, USA) equipped with an Autospec-Premier mass spectrometer (Waters Corp., Milford, MA, USA). Specifically, congeners of seven PCDDs, ten PCDFs, and four non-*ortho* PCBs were analysed according to previously published methods^16–18^. The dioxin detection limits per lipid weight were as follows: PCDDs and PCDFs, 0.3–2 pg/g lipid for umbilical cord blood, placenta, vernix caseosa, meconium, and amniotic fluid and 1–4 pg/g lipid for maternal blood; non-*ortho* PCBs, 0.3–0.6 pg/g lipid for placenta, 0.3–1 pg/g lipid for umbilical cord blood, placenta, vernix caseosa, meconium, and amniotic fluid and 10 pg/g lipid for maternal blood.

As the amounts of vernix caseosa and meconium obtained during delivery were limited, reproducibility tests using these specimens were not carried out in the present study. We identified that the recovery of the clean-up spike for each sample ranged from 50% to 120% according to the provisional manual for blood dioxin analysis issued by the Ministry of Health, Labour, and Welfare, Japan (2001). A reference serum sample was analysed for every batch, and analytical values fell within the predefined range.

To evaluate the accuracy and reliability of dioxin analysis, quality control studies on blood as a typical biological human tissue were completed once every year, and the results were compared against those obtained at three other laboratories in Japan. The average variation among the dioxin concentrations in human blood samples was considered acceptable if it was within 10%.

Because the toxic equivalent quantity (TEQ) has been proven a valuable measure in multiple studies on dioxins^19^, TEQs were calculated by multiplying the levels of individual congeners by their TEF value, as recommended by the World Health Organization in 2005^206^, and concentrations below the detection limit were designated as zero^18–21^.
